# Supplementary material for: Human ACE2 peptide-mimics block SARS-CoV-2 pulmonary cells infection
Source: Commun Biol. 2021 Feb 12;4:197. doi: 10.1038/s42003-021-01736-8 (PMC7881012; doi:10.1038/s42003-021-01736-8)
Supplement: Supplementary file 2 — Supplementary Information [file 42003_2021_1736_MOESM2_ESM.pdf]

Human ACE2 peptide-mimics block SARS-CoV-2 Pulmonary Cells Infection

Philippe Karoyan,[1, 2, 3]* Vincent Vieillard,[4] Luis Gómez-Morales,[1, 2] Estelle Odile,[1, 2] Amélie Guihot,[5] Charles-Edouard Luyt,[6] Alexis Denis,[7] Pascal Grondin,[7] Olivier Lequin[1]

[1]Sorbonne Université, École Normale Supérieure, PSL University, CNRS, Laboratoire des Biomolécules, LBM, 75005 Paris, France.

[2]Sorbonne Université, École Normale Supérieure, PSL University, CNRS, Laboratoire des Biomolécules, LBM, Site OncoDesign, 25-27 Avenue du Québec, 91140 Villebon Sur Yvette, France.

[3] χ-Pharma, 25 avenue du Québec, 91140 Villebon Sur Yvette, France.

[4]Sorbonne Université, INSERM, CNRS, Centre d'Immunologie et des Maladies Infectieuses-Paris (CIMI-Paris), F-75013 Paris, France.

[5]Assistance Publique-Hôpitaux de Paris (AP-HP), Hôpital Pitié-Salpêtrière, Département d'Immunologie, F-75013, Paris, France. Sorbonne Université, Inserm U1135, Centre d'Immunologie et des Maladies Infectieuses, CIMI-Paris, F-75013, Paris, France.

[6]Assistance Publique–Hôpitaux de Paris (AP-HP), Hôpital Pitié–Salpêtrière, Service de Médecine Intensive Réanimation, Institut de Cardiologie, F-75013, Paris, France.

[7]Oncodesign, 25 Avenue du Québec, 91140 Villebon Sur Yvette, France.

* Correspondance : philippe.karoyan@sorbonne-universite.fr.

Phone: +33 1 44276237

SUPPLEMENTARY INFORMATION

Supplementary Tables

Supplementary Table 1

**Supplementary table 1. Residues involved in ACE2 / SARS-CoV2 Spike interaction**				
ACE2	Residue	Interactions with SARS-CoV-2 Spike residues		
		Hydrogen bond	Salt bridge	Van der Waals
H1	Q24	N487 (OE1–ND2 2.7 Å)		
	T27			F456, A475, Y489
	F28			Y489
	D30	K417 (OD2–NZ 2.9 Å)	K417 (OD2/OD1–NZ 2.9/4.0 Å)	
	K31	Q493 (NZ–NE2 2.9 Å)	E484 (NZ–OE1 4.4 Å)	F456, Y489
	H34			Y453, L455
	E35	Q493 (OE1/OE2–NE2 3.1/3.5 Å)		
	E37	Y505 (OE2–OH 3.5 Å)		
	D38	Y449 (OD2/OD1–OH 2.7/3.2 Å)		
	Y41	T500 (OH–OG1 2.7 Å)<br>N501 (OH–N 3.7 Å)		Q498
	Q42	G446 (NE2–O 3.2 Å)<br>Y449 (NE2–OH 2.8 Å)<br>Q498 (OE1–NE2 3.4 Å)		
	L45			Q498, T500
H2	L79			F486
	M82			F486
	Y83	N487 (OH–OD1 2.8 Å)<br>Y489 (OH–OH 3.5 Å)		F486
	N330			T500
	K353	G496 (NZ–O 3.1 Å)<br>G502 (O–N 2.8 Å)		Y505
	G354			G502
	D355	T500 (OD2–O 3.3 Å)		
	R357	T500 (NH1–OG1 3.7 Å)		
	R393	Y505 (NH2–OH 3.7 Å)		
H1 and H2, Helix 1 and 2; ND2, nitrogen delta 2; NE2, nitrogen epsilon 2; NZ, nitrogen zeta; N, nitrogen; NH1, nitrogen eta 1: NH2, nitrogen eta 2; OH, oxygen eta; O, oxygen; OD1, oxygen delta 1; OD2, oxygen delta 2; OG1, oxygen gamma 1; OE1, oxygen epsilon 1; OE2, oxygen epsilon 2.				

Supplementary Table 2

Supplementary Table 2. **Agadir calculation of the helical content of the 27 to 29-mers peptide mimics.**

**PEPTIDE SEQUENCE**	**PREDICTED HELICAL CONTENT [a] %**
STIEEQAKTFLDKFNHEAEDLFYQSSL-NH_2	6
STIEEQAKTFLDKFLHEAEDLFYQSSL-NH_2	8
STIEEQLKTFLDKFLHEAEDLFYQSSL-NH_2	21
STIEEQLKTFLDKFLHELEDLFYQSSL-NH_2	33
SLIEEQLKTFLDKFLHELEDLFYQSSL-NH_2	38
STLEEQLKTFLDKFLHELEDLFYQSSL-NH_2	36
SLAEEQLKTFLDKFLHELEDLFYQSSL-NH_2	38
SALEEQLKTFLDKFLHELEDLFYQSSL-NH_2	44
SALEEQLKTFLDKFLHELEDLFYQASL-NH_2	45
SALEEQLKTFLDKFLHELEDLFYQSAL-NH_2	44
SALEEQLKTFLDKFLHELEDLFYQAAL-NH_2	49
SALAEQLKTFLDKFLHELEDLFYQASL-NH_2	30
SALEEQLATFLDKFLHELEDLFYQASL-NH_2	24
SALEEQLKTFLDKALHELEDLFYQASL-NH_2	42
SALEEQLKTFLDKFLHELEDLAYQASL-NH_2	51
SALEEQLKTFLDKFLHELEDLAYQSSL-NH_2	47
SALEEQLKTFLDKFLHELEDLAYQLSL-NH_2	54
SALEEQLKTFLDKFLHELEDLLYQASL-NH_2	55
SLLEEQLKTFLDKFLHELEDLAYQASL-NH_2	47
SALEEQLKTFLDKFLHELEDLLYQLSL-NH_2	64
SALEEQLKTFLDKFLHELEDLLYQLAL-NH_2	78
Ac*-SALEEQLKTFLDKFLHELEDLLYQLAL-NH_2	66
SAIEEQLKTFLDKFLHELEDLLYQLAL-NH_2	76
SVLEEQLKTFLDKFLHELEDLLYQLAL-NH_2	70
SILEEQLKTFLDKFLHELEDLLYQLAL-NH_2	71
SALEEQLKTFLDKFLHELEPLLYQLAL-NH_2	32
SALEEQLKTFLDKFLHELEDPLYQLAL-NH_2	34
STIEEQAKTFLDKFNHEAEDLFYQSSLAS-NH_2<br>Native sequence 29-mer	5
SALEEQLKTFLDKFLHELEDLLYQLALAS-NH_2<br>29-mer	79
SALEEQLKTFLDKFLHELEDLLYQLALAL-NH_2<br>29-mer	88
SALEEQYKTFLDKFLHELEDLLYQLALAL-NH_2<br>29-mer	84
SALEEQ*h*YKTFLDKFLHELEDLLYQLALAL-NH_2<br>29-mer	NA
AHLFSYLTTKEEQDNDAIFLQEFSKES-NH_2<br>(scrambled [b])	1
IEEQAKTFLDKFNHEAEDLFYQS-NH_2<br>(Ppen SBP1-derived sequence)	1

[a] The calculation of the helical content was realized for peptides fully protected or deprotected at the *N*-and *C*-termini, together with partial *N*- or *C*-protection. Only the values for the *C*-carboxamide peptides are here reported, excepted when Ac*- is indicated.
[b] Scrambled peptide designed from native helix sequence using a scrambler tool[1]
*h*Y, homotyrosine; NA, not applicable (for *h*Tyr containing sequences)

Supplementary Table 3

**Supplementary Table 3. Antigenicity prediction using the semi-empirical method reported by Kolaskar and Tongaonkar.**		
**PEPTIDE SEQUENCE**	**HELICAL CONTENT%**	ANTIGENICITY (AD)
STIEEQAKTFLDKFNHEAEDLFYQSSL-NH_2	6	0
STIEEQAKTFLDKFLHEAEDLFYQSSL-NH_2	8	1
STIEEQAKTFLDKFMHEAEDLFYQSSL-NH_2	7	0
SALEEQLKTFLDKFLHELEDLLYQLSL-NH_2	64	1
SALEEQLKTFLDKFNHELEDLLYQLSL-NH_2	30	0
SALEEQLKTFLDKFMHELEDLLYQLSL-NH_2	54	0
SALEEQ(*h*Y)*KTFLDKFMHELEDLLYQLSL-NH_2	NA	NA
SALEEQLKTFLDKFLHELEDLLYQLAL-NH_2	78	1
SALEEQLKTFLDKFNHELEDLLYQLAL-NH_2	38	0
SALEEQLKTFLDKFMHELEDLLYQLAL-NH_2	68	0
SALEEQ(*h*Y)KTFLDKFMHELEDLLYQLAL-NH_2	NA	NA
SAIEEQLKTFLDKFLHELEDLLYQLAL-NH_2	76	1
SAIEEQLKTFLDKFNHELEDLLYQLAL-NH_2	36	0
SAIEEQLKTFLDKFMHELEDLLYQLAL-NH_2	67	0
SALEEQLKTFLDKFLHELEDPLYQLAL-NH_2	34	1
SALEEQLKTFLDKFNHELEDPLYQLAL-NH_2	23	0
SALEEQLKTFLDKFMHELEDPLYQLAL-NH_2	30	0
SALEEQLKTFLDKFLHELEDLLYQLALAS-NH_2	79	1
SALEEQLKTFLDKFMHELEDLLYQLALAS-NH_2	73	1
SALEEQLKTFLDKFLHELEDLLYQLALAL-NH_2	88	1
SALEEQLKTFLDKFNHELEDLLYQLALAL-NH_2	54	1
SALEEQLKTFLDKFMHELEDLLYQLALAL-NH_2	83	1
AHLFSYLTTKEEQDNDAIFLQEFSKES-NH_2 (scrambled)	1	0
IEEQAKTFLDKFNHEAEDLFYQS-NH_2 (Ppen, SBP1-derived sequence)	1	0
AD, antigenic determinants; *h*Y, homotyrosine; NA, not applicable (for hTyr containing sequences).		

Supplementary Table 4

Supplementary Table 4. Sequences and physico-chemical properties of synthesized peptides

Code P	Sequence [a]	Predicted helical content% [b]	Mean Hydrophobicity [c]	Hydrophobic moment [c]	Net charge
**P1**	STIEE QAKTF LDKFN HEAED LFYQS SL-NH_2	6	0.27	0.29	-4
**P1scr**	AHLFS YLTTK EEQDN DAIFL QEFSK ES-NH_2	1	0.27	0.03	-4
**P2**	SALEE QLKTF LDKFL HELED LLYQL SL-NH_2	64	0.52	0.44	-4
**P3**	SALEE QLKTF LDKFL HELED LLYQL AL-NH_2	78	0.53	0.44	-4
**P4**	Ac-SALEE QLKTF LDKFL HELED LLYQL AL-NH_2	66	0.53	0.44	-4
**P5**	SALEE QLKTF LDKFL HELED PLYQL AL-NH_2	34	0.49	0.41	-4
**P6**	SALEE QLKTF LDKFL HELED LLYQL ALAL-NH_2	88	0.56	0.46	-4
**P7**	SALEE QYKTF LDKFL HELED LLYQL ALAL-NH_2	NA	0.54	0.43	-4
**P8**	SALEE QLKTF LDKFM HELED LLYQL AL-NH_2	68	0.51	0.44	-4
**P9**	SALEE QYKTF LDKFM HELED LLYQL SL-NH_2	NA	0.47	0.41	-4
**P10**	SALEE QYKTF LDKFM HELED LLYQL AL-NH_2	NA	0.48	0.41	-4
**Ppen**	IEE QAKTF LDKFN HEAED LFYQS-NH_2	1	0.23	0.41	-4

[a] Homotyrosine residue is depicted with underlined letter Y.
[b] Predicted helical content using Agadir program; NA, not applicable (for homotyrosine containing sequences).
[c] Mean Hydrophobicity and hydrophobic moment were computed with HELIQUEST program.[2]

Supplementary Table 5

Supplementary Table 5. Synthesized peptide mimics analyses.

Code (HPLC [a])	Code P	Sequence (Chemical Formula [b])	MW	m/z calculated [b] EZ+	m/z observed EZ+	m/z calculated EZ-	m/z observed EZ-	RT (min)	Purity
12 (B)	**Ppen**	IEEQAKTFLDKFNHEAEDLFYQS-NH$_2$<br>($C_{127}H_{185}N_{31}O_{41}$)	2802,05	1402 (M+2H$^+$)<br>934,8 M+3H$^+$)<br>701,4 (M+4H$^+$)	1402,3 (M+2H$^+$)<br>935,2 (M+3H$^+$)<br>701,6 (M+4H$^+$)	1400,0 (M-2H$^+$)<br>933,0 (M-3H$^+$)<br>699,5 (M-4H$^+$)	1400,1 (M-2H$^+$)<br>933,2 (M-3H$^+$)	1,62	>95%
1 (B)	**P1**	STIEEQAKTFLDKFNHEAEDLFYQSSL-NH$_2$<br>($C_{143}H_{213}N_{35}O_{48}$)	3190,47	1596,2 ((M+2H$^+$)<br>1064,7(M+3H$^+$)<br>798,7 (M+4H$^+$)	1596,6 (M+2H$^+$)<br>1064,7(M+3H$^+$)<br>798,7 (M+4H$^+$)	1594,2 (M-2H$^+$)<br>1062,5 (M-3H$^+$)<br>796,6 (M-4H$^+$)	1594,5 (M-2H$^+$)<br>1063,5(M-3H$^+$)<br>796,8 (M-4H$^+$)	1,84	>95%
11 (B)	**P1scr**	AHLFSYLTTKEEQDNDAIFLQEFSKES-NH$_2$<br>($C_{143}H_{213}N_{35}O_{48}$)	3190,47	1596,2 ((M+2H$^+$)<br>1064,7(M+3H$^+$)<br>798,7 (M+4H$^+$)	1596,6 (M+2H$^+$)<br>1064,7 (M+3H$^+$)<br>798,7 (M+4H$^+$)	1594,3 (M-2H$^+$)<br>1062,5 (M-3H$^+$)<br>796,6 (M-4H$^+$)	1594,6 (M-2H$^+$)<br>1063 (M-3H$^+$)<br>797,3 (M-4H$^+$)	1,54	>90%
2 (B)	**P2**	SALEEQLKTFLDKFLHELEDLLYQLSL-NH$_2$<br>($C_{150}H_{236}N_{34}O_{45}$)	3235,73	1618,8 (M+2H$^+$)<br>1079,6 (M+3H$^+$)<br>810,0 (M+4H$^+$)	1619,2 (M+2H$^+$)<br>1079,8 (M+3H$^+$)<br>810.0 (M+4H$^+$)	1616,8 (M-2H$^+$)<br>1077,6 (M-3H$^+$)<br>807,9 (M-4H$^+$)	1616,6 (M-2H$^+$)<br>1077,8 (M-3H$^+$)	1.47	>95%
3 (B)	**P3**	SALEEQLKTFLDKFLHELEDLLYQLAL-NH$_2$<br>($C_{150}H_{236}N_{34}O_{44}$)	3219,73	1610,8 (M+2H$^+$)<br>1074,2 (M+3H$^+$)<br>805,9 (M+4H$^+$)	1611,1 (M+2H$^+$)<br>1074,4 (M+3H$^+$)<br>806 (M+4H$^+$)	1608,8 (M-2H$^+$)<br>1072,2 (M-3H$^+$)<br>803,9 (M-4H$^+$)	1608,9 (M-2H$^+$)<br>1072,2 (M-3H$^+$)	2,53	>95%
4 (B)	**P4**	Ac-SALEEQLKTFLDKFLHELEDLLYQLAL-NH$_2$<br>($C_{152}H_{238}N_{34}O_{46}$)	3261,77	1631,9 (M+2H$^+$)<br>1088,2 (M+3H$^+$)<br>816,4 (M+4H$^+$)	1632,2 (M+2H$^+$)<br>1088,5 (M+3H$^+$)<br>816,5 (M+4H$^+$)	1629,9 (M-2H$^+$)<br>1086,2 (M-3H$^+$)<br>814,4 (M-4H$^+$)	1630,3 (M-2H$^+$)<br>1086,5 (M-3H$^+$)	2,69	>95%
5 (B)	**P5**	SALEEQLKTFLDKFLHELEDPLYQLAL-NH$_2$<br>($C_{149}H_{232}N_{34}O_{44}$)	3203,69	1602,8 (M+2H$^+$)<br>1068,9 (M+3H$^+$)<br>801,9 (M+4H$^+$)	1603,2 (M+2H$^+$)<br>1069,1 (M+3H$^+$)<br>802,0 (M+4H$^+$)	1601,3 (M-2H$^+$)<br>1067,2 (M-3H$^+$)<br>800,2 (M-4H$^+$)	1601,6 (M-2H$^+$)<br>1067,1 (M-3H$^+$)	2,16	>95%
6 (B)	**P6**	SALEEQLKTFLDKFLHELEDLLYQLALAL-NH$_2$<br>($C_{159}H_{252}N_{36}O_{46}$)	3403,97	1702,98 (M+2H$^+$)<br>1135,65 (M+3H$^+$)<br>851,99 (M+4H$^+$)	1703,3 (M+2H$^+$)<br>1135,9 (M+3H$^+$)<br>852,1 (M+4H$^+$)	1701,5 (M-2H$^+$)<br>1133,9 (M-3H$^+$)<br>850,2 (M-4H$^+$)	1701,7 (M-2H$^+$)<br>1133,8 (M-3H$^+$)	2,74	>95%
9 (B)	**P7**	SALEEQ*h*YKTFLDKFLHELEDLLYQLALAL-NH$_2$<br>($C_{163}H_{252}N_{36}O_{47}$)	3468,01	1735 (M+2H$^+$)<br>1157 (M+3H$^+$)<br>868 (M+4H$^+$)	1735,2 (M+2H$^+$)<br>1157,2 (M+3H$^+$)<br>868,1 (M+4H$^+$)	1733 (M-2H$^+$)<br>1155 (M-3H$^+$)<br>866 (M-4H$^+$)	1733,3 (M-2H$^+$)<br>1155,3 (M-3H$^+$)	2,57	>95%
13 (B)	**P8**	SALEEQLKTFLDKFMHELEDLLYQLAL-NH$_2$<br>($C_{149}H_{234}N_{34}O_{44}S$)	3237,77	1619,9 (M+2H$^+$)<br>1080,3 (M+3H$^+$)<br>810,4 (M+4H$^+$)	1620,2 (M+2H$^+$)<br>1080,4 (M+3H$^+$)<br>810,5 (M+4H$^+$)	1617,9 (M-2H$^+$)<br>1078,3 (M-3H$^+$)<br>808,4 (M-4H$^+$)	1618,9 (M-2H$^+$)<br>1078,1 (M-3H$^+$)	2,45	>95%
15 (B)	**P9**	SALEEQ(hY)KTFLDKFMHELEDLLYQLSL-NH$_2$<br>($C_{153}H_{234}N_{34}O_{46}S$)	3317,81	1659,9 (M+2H$^+$)<br>1106,9 (M+3H$^+$)<br>830,4 (M+4H$^+$)	1660,2 (M+2H$^+$)<br>1107,2(M+3H$^+$)<br>830,6 (M+4H$^+$)	1657,9 (M-2H$^+$)<br>1104,9 (M-3H$^+$)<br>828,4 (M-4H$^+$)	1658,0 (M-2H$^+$)<br>1105,1 (M-3H$^+$)<br>(M-4H$^+$)	2,31	>95%
14 (B)	**P10**	SALEEQ(*h*Y)KTFLDKFMHELEDLLYQLAL-NH$_2$<br>($C_{153}H_{234}N_{34}O_{45}S$)	3301,81	1651,9 (M+2H$^+$)<br>1101,6 (M+3H$^+$)<br>826,4 (M+4H$^+$)	1652,2 (M+2H$^+$)<br>1101,8 (M+3H$^+$)<br>826,6 (M+4H$^+$)	1649,9 (M-2H$^+$)<br>1099,6 (M-3H$^+$)<br>824,4 (M-4H$^+$)	1650,1 (M-2H$^+$)<br>1099,9 (M-3H$^+$)	2,34	>95%

[a] HPLC method as described in the main Methods
[b] calculated with ChemDraw software
MW, molecular weight; m/z, mass to charge ratio; RT, HPLC retention time.

Supplementary Table 6

Table 6. Octet-RED BLI analysis

	Sample ID	Conc. (nM)	Response	K_D (M)	K_D Error	ka (Ms^{-1})	ka Error	kdis (s^{-1})	kdis Error	Full R^2
EXP1	**ACE2**	10	0.3118	3.94E-10	5.63E-12	15.78E+04	4.79E+02	6.22E-05	8.68E-07	0.9989
EXP2	**ACE2**	100	1.0175	4.13E-10	2.66E-11	8.00E+04	2.50E+02	3.30E-05	2.12E-06	0.9801
EXP3	**ACE2**	100	0.6917	5.72E-10	2.33E-11	8.97E+04	2.77E+02	5.13E-05	2.08E-06	0.9783
EXP1	**P8**	100	0.0218	1.59E-08	1.26E-09	5.61E+03	5.95E+03	8.94E-05	6.99E-06	0.8764
EXP2	**P8**	1000	0.0191	3.17E-08	2.02E-09	4.31E+03	6.10E+01	1.37E-04	8.47E-06	0.8687
EXP1	**P9**	1000	0.0209	4.74E-11	5.40E-10	1.02E+03	3.31E+01	<1.0E-07	5.50E-07	0.9304
EXP2	**P9**	1111	0.0203	4.38E-11	4.50E-10	1.10E+03	2.71E+01	<1.0E-07	4.95E-07	0.9381
EXP3	**P9**	10000	0.0406	1.90E-10	1.48E-09	2.54E+02	2.50E+00	<1.0E-07	3.76E-07	0.9608
EXP1	**P10**	1000	0.046	1.94E-11	1.39E-10	2.49E+03	2.28E+01	<1.0E-07	3.45E-07	0.9697
EXP2	**P10**	1111	0.0282	3.43E-11	4.19E-10	1.41E+03	3.29E+01	<1.0E-07	5.91E-07	0.9248
EXP3	**P10**	1000	0.0446	3.36E-11	3.70E-10	1.43E+03	3.27E+01	<1.0E-07	5.31E-07	0.9422

K_D, equilibrium dissociation constant; ka, association constant; kdis, dissociation constant; R^2, coefficient of determination.

<u>**Supplementary Figures**</u>

Supplementary Figure 1

a) **PNITNLCPFGEVFNATRFASVYAWNRKRISNCVADYSVLYNSASFSTFKCYGVSPTKLNDLCFTNVYADSFVI**
RGDEVRQIAPGQTGKIADYNYKLPDDFTGCVIAWNSNNLDSKVGGNYNYLYRLFRKSNLKPFERDISTEIYQ
AGSTPCNGVEGFNCYFPLQSYGFQPTNGVGYQPYRVVVLSFELLHAPATVEPKSCDKTHTCPPCPAPELLG
GPSVFLFPPKPKDTLMISRTPEVTCVVVDVSHEDPEVKFNWYVDGVEVHNAKTKPREEQYNSTYRVVSVLTVL
HQDWLNGKEYKCKVSNKALPAPIEKTISKAKGQPREPQVYTLPPSRDELTKNQVSLTCLVKGFYPSDIAVEWES

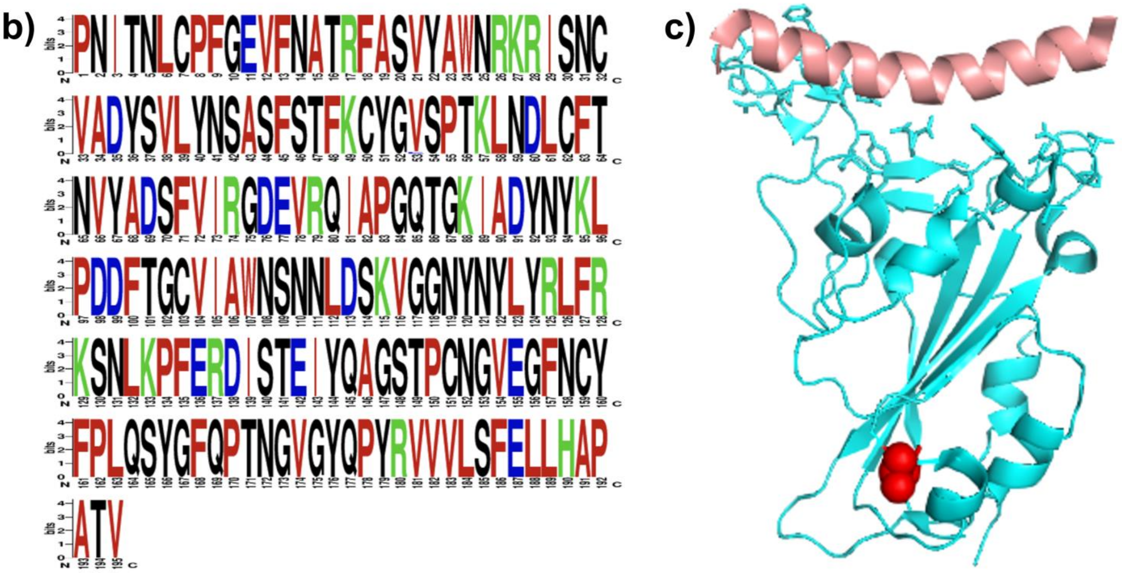

Supplementary Figure 1. **Highly conserved sequence of the SARS-CoV-2 RBD motif. a)** Complete sequence of the SARS-CoV-2-huFc fusion protein used in the present study (Sanyou Bio, reference PN003). The RBD motif is highlighted in bold, the rest represent the huFc fragment. **b)** Sequence alignment of 24 random RBD amino acid sequences randomly listed on NCBI resources from China (QOH25833, QIG55857, QHR63290, QHR63250, QJG65957, QJG65956, QJG65951), USA (QIV65044, QJD23847, QJU11481, QJD24531, QJD25193, QJD25529, QJA17180), and France (QJT73034, QJT73010, QJT72902, QJT72806, QJT72794, QJT72722, QJT72710, QJT72626, QJT72614, QJT72554), and the RBD motif from the SARS-CoV-2-huFc fusion protein. The alignment was performed using the Weblogo software (http://weblogo.berkeley.edu/) and validated using Clustal Omega software. Both confirmed one point-mutation (V53E) in one (QJT72806) out of the 24 sequences. **c)** Structure of the complex between hACE2 and the Spike protein of SARS-CoV-2 (pdb 6m0j) highlighting the V53 side chain in red sphere.

Supplementary Figure 2

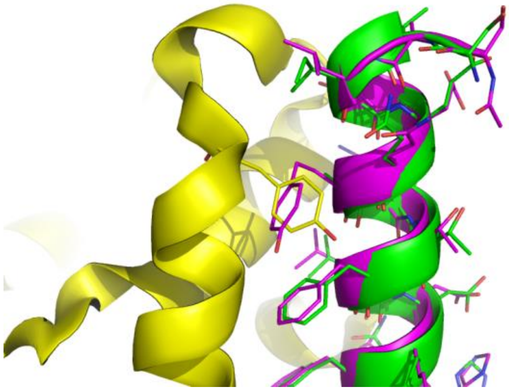

Supplementary Figure 2. Superposition of ACE2 protein with a modelled ACE2 H1 helix (shown in magenta) incorporating a *h*Tyr residue in position 25. Helix H1 of ACE2 is shown in green and other parts of the protein (including residue Y83 in helix H2) in yellow. The modelled structure was calculated with Amber 14 program and ff14SB forcefield using a restrained molecular dynamics protocol, as described.[3] Distance and dihedral angle restraints were defined from ACE2 H1 helix template to constraint hydrogen bonds and $\phi, \psi, \chi 1$ dihedral angles in the calculated structure.

Supplementary Figure 3

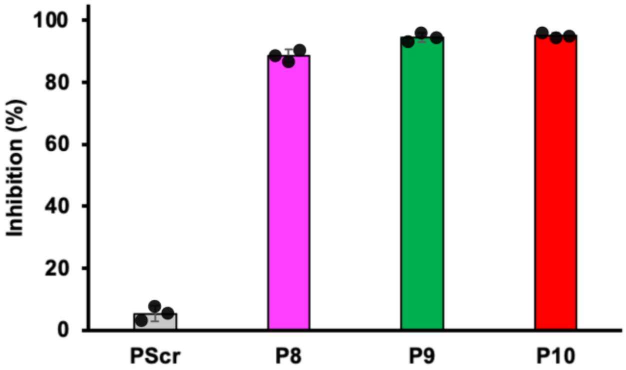

Supplementary Figure 3. Inhibition of SARS-CoV-2 replication in Vero-E6 cells using P9 and P10. Vero-E6 cells were infected with SARS-CoV-2/PSL2020 P#2 stock at a multiplicity of infection (MOI) of 0.1 in the presence of **P9** and **P10** (**P8** and **P1scr** were used as controls) at 10 µM for 2 h. Then, the virus was removed, and cultures were washed, incubated for 48 h, before supernatant was collected to measure virus replication by ELISA. Histograms represent the means of three independent experiments (each performed by duplicate; dots above). Data is expressed as compared to untreated SARS CoV-2-infected Vero-E6 cells

Supplementary References

1. Peptide scrambling tool available at peptidenexus.com/article/sequence-scrambler
2. Gautier, R., Douguet, R., Antonny, B. & Drin, G. HELIQUEST: a web server to screen sequences with specific a-helical properties. *Bioinformatics* 24, 2101-2 (2008)
3. Byrne, C., Belnou, M., Baulieu, E. E., Lequin, O. & Jacquot, Y. Electronic circular dichroism and nuclear magnetic resonance studies of peptides derived from the FKBP52-interacting beta-turn of the hERalpha ligand-binding domain. *Pept. Sci*., e24113 (2019).
4. Wrapp, D.; Wang, N.; Corbett, K.S.; Goldsmith, J.A.; Hsieh, C-L.; Abiona, O.; Graham, B.S.; McLellan, J.S. Cryo-EM structure of the 2019-nCoV spike in the prefusion conformation, *Science* (2020)
