# Supplementary material for: Human ACE2 peptide-mimics block SARS-CoV-2 pulmonary cells infection
Source: Commun Biol. 2021 Feb 12;4:197. doi: 10.1038/s42003-021-01736-8 (PMC7881012; doi:10.1038/s42003-021-01736-8)
Supplement: Supplementary file 3 — Description of Additional Supplementary Files [file 42003_2021_1736_MOESM3_ESM.pdf]

Description of Additional Supplementary Items

File Name: Supplementary Data 1

Description: LC-MS spectra of P1-10, Pscr and Ppen

File Name: Supplementary Data 2

Description: Materials and buffers used for BLI experiments with 2 their corresponding suppliers and reference codes

File Name: Supplementary Data 3

Description: Source data for Fig 3.
