# Supplementary figures and images for: Human ACE2 peptide-mimics block SARS-CoV-2 pulmonary cells infection
Source: Commun Biol. 2021 Feb 12;4:197. doi: 10.1038/s42003-021-01736-8 (PMC7881012; doi:10.1038/s42003-021-01736-8)

**Supplementary Data 1: LC-MS spectra of P1-10, Pscr and Ppen**

**P1**


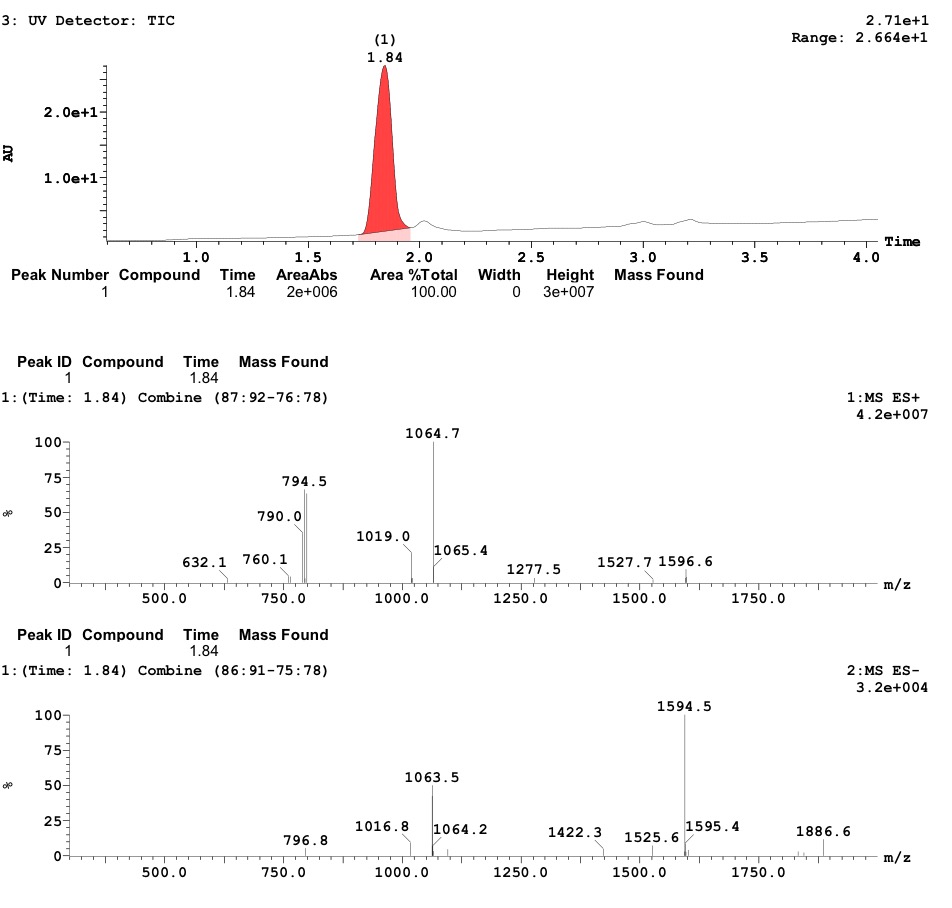


**P2**


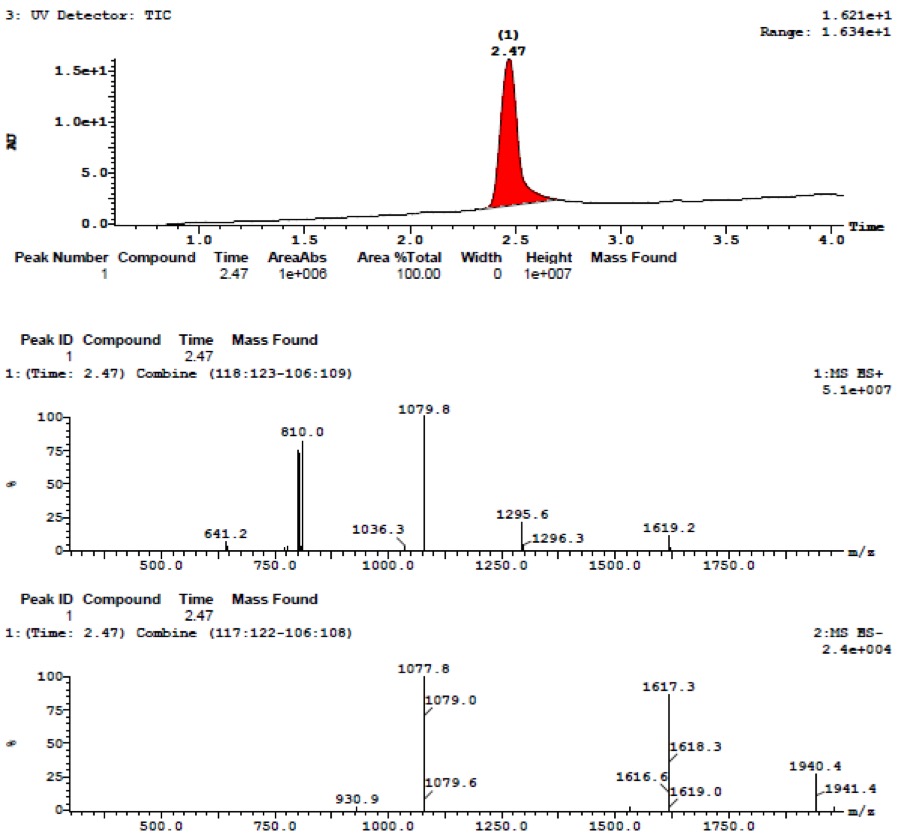


**P3**


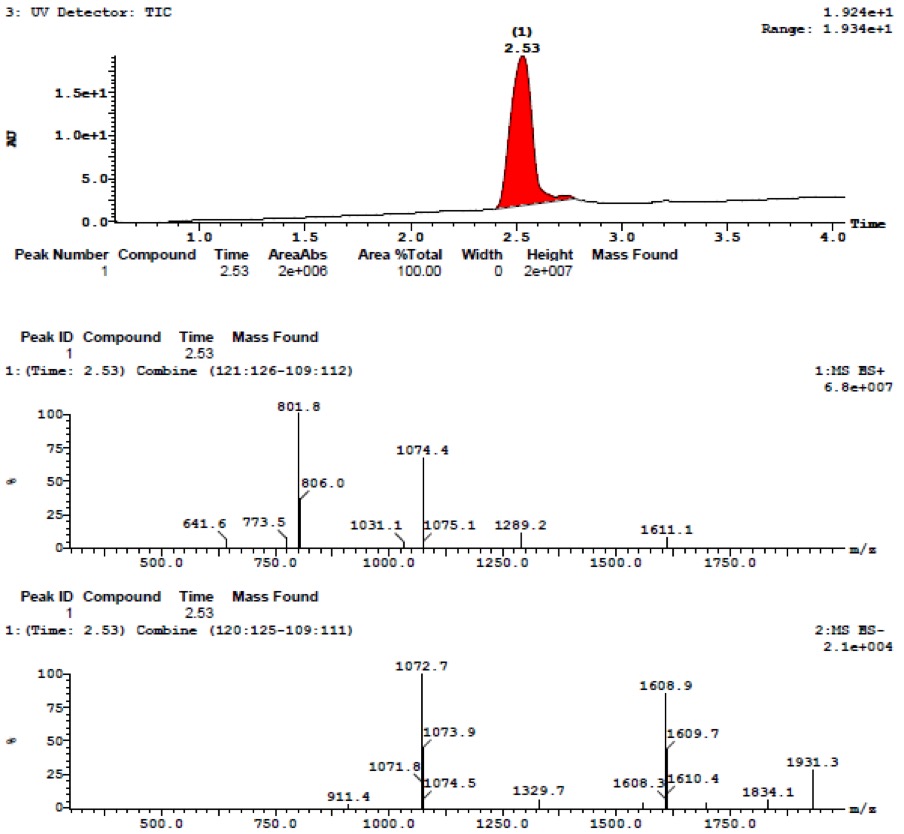


**P4**


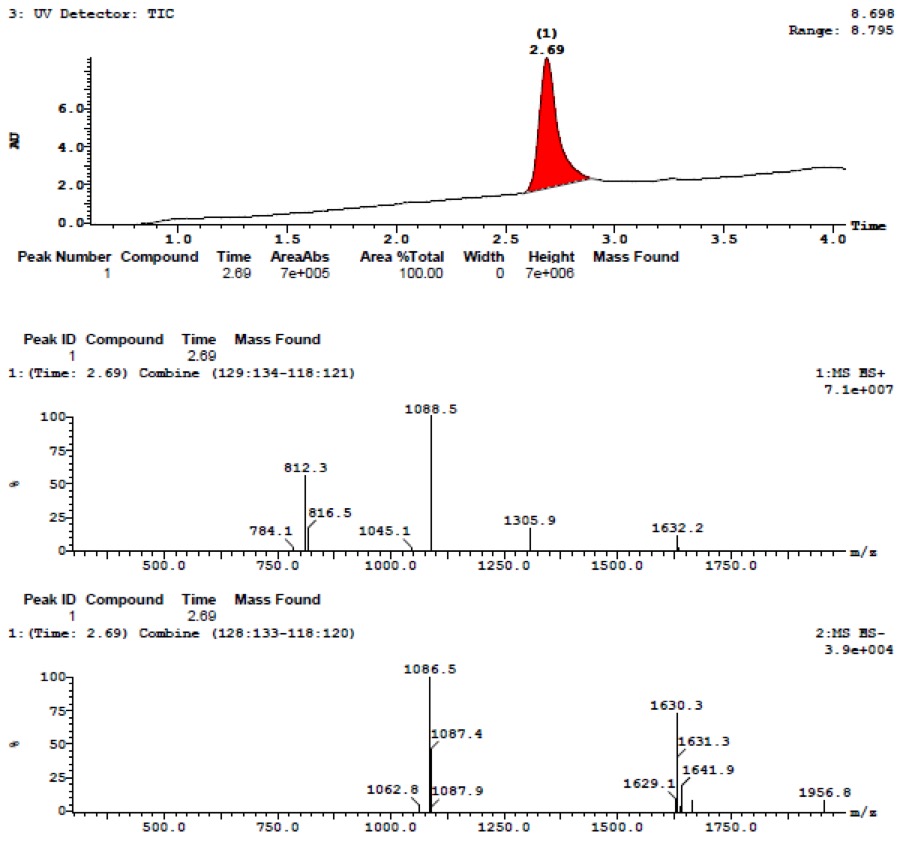


**P5**


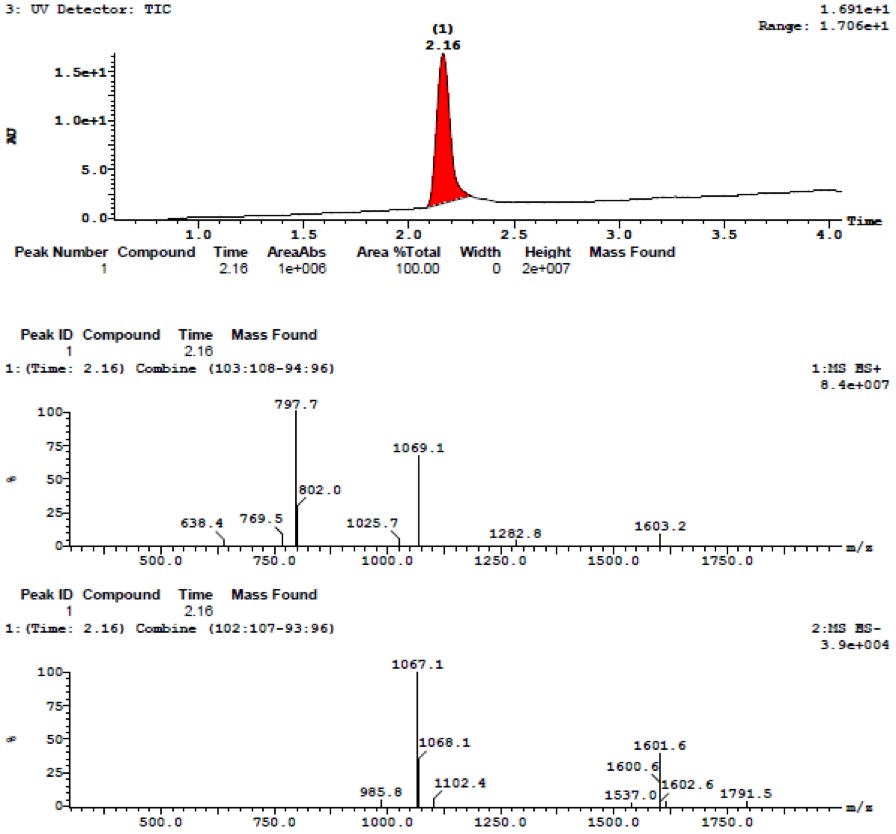


**P6**


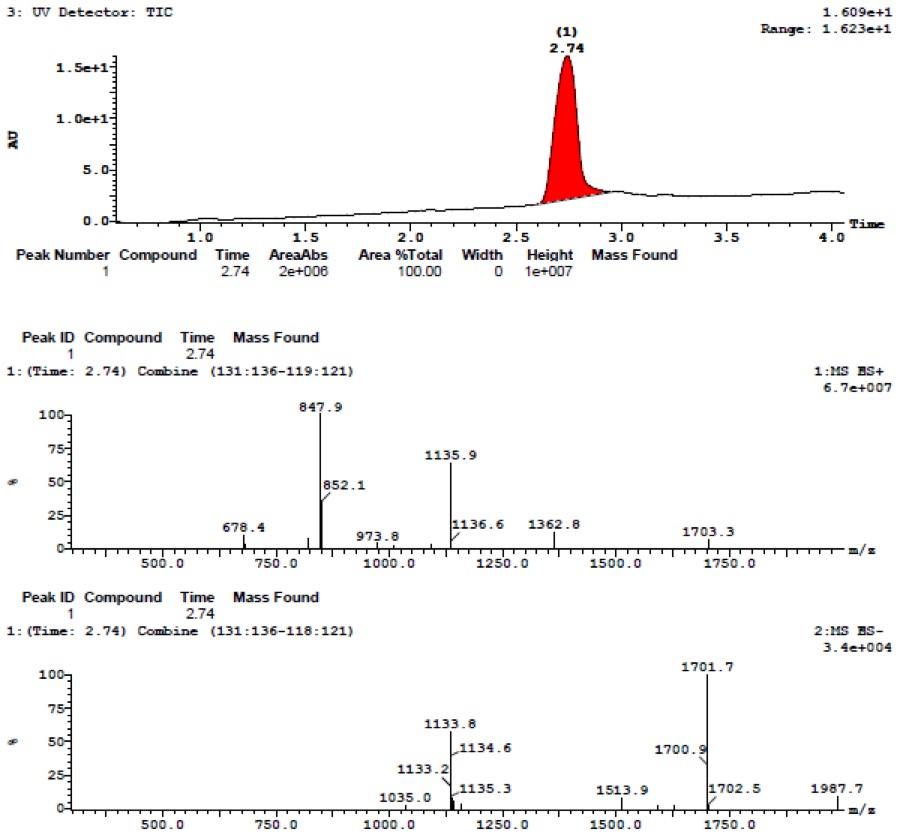


**P7**


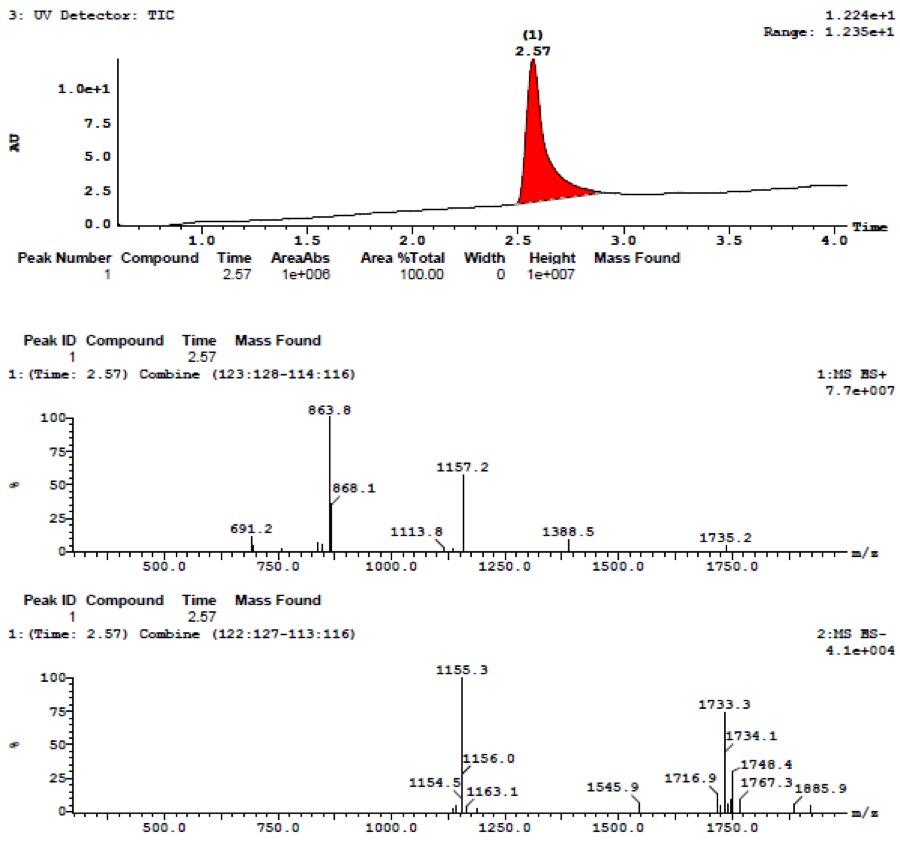


**P8**


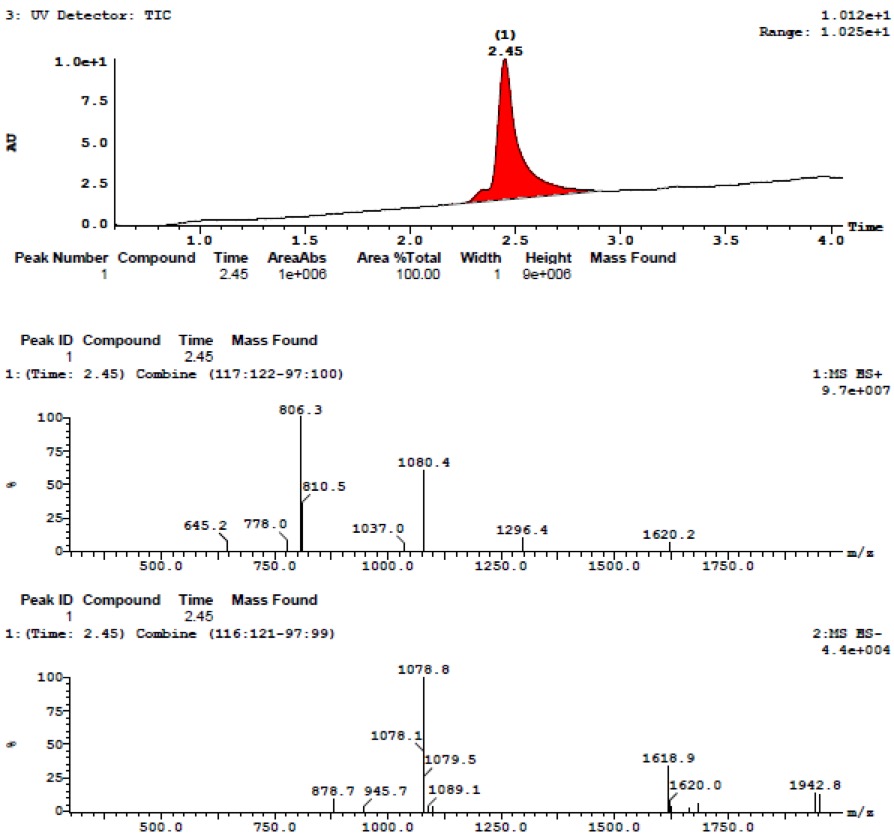


**P9**


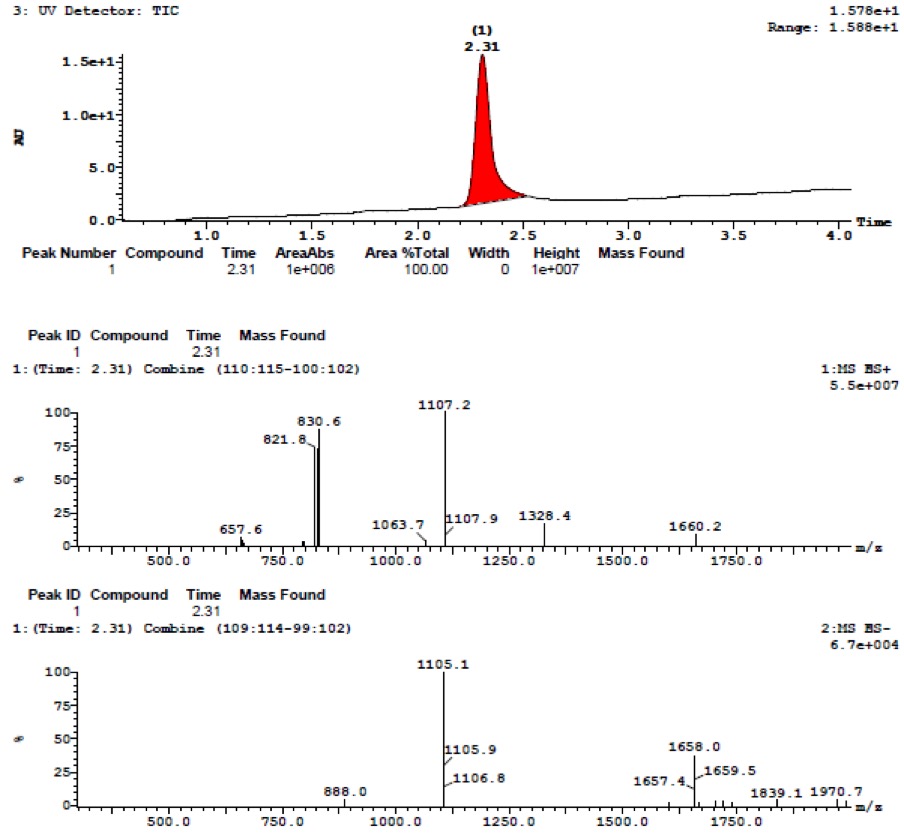


**P10**


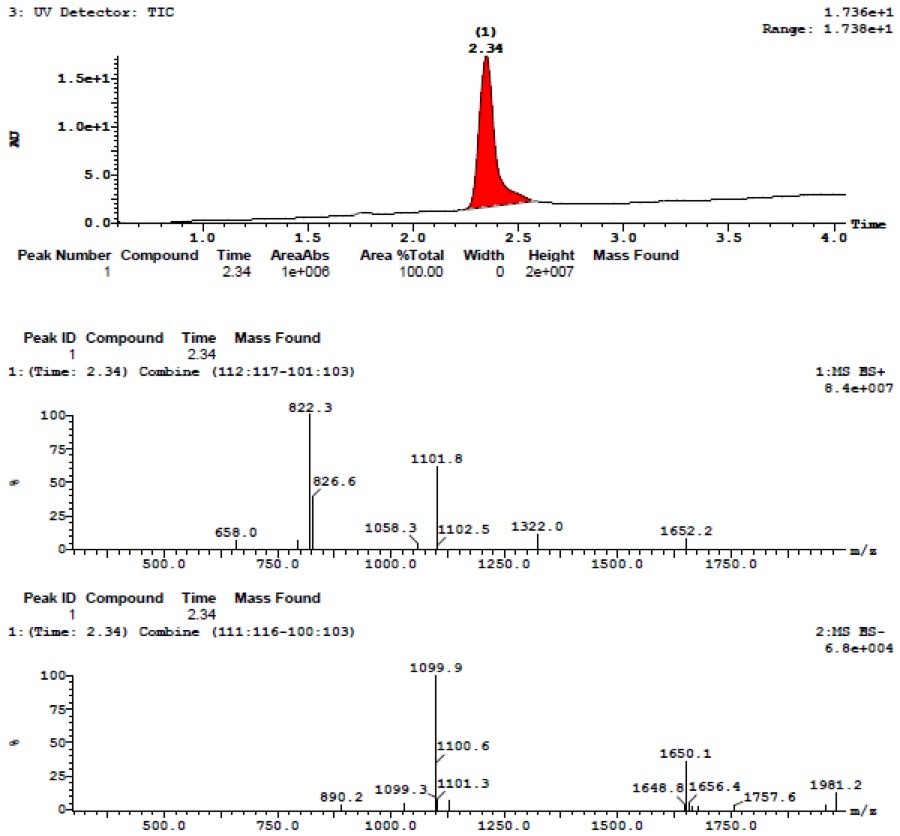


**Pscr**


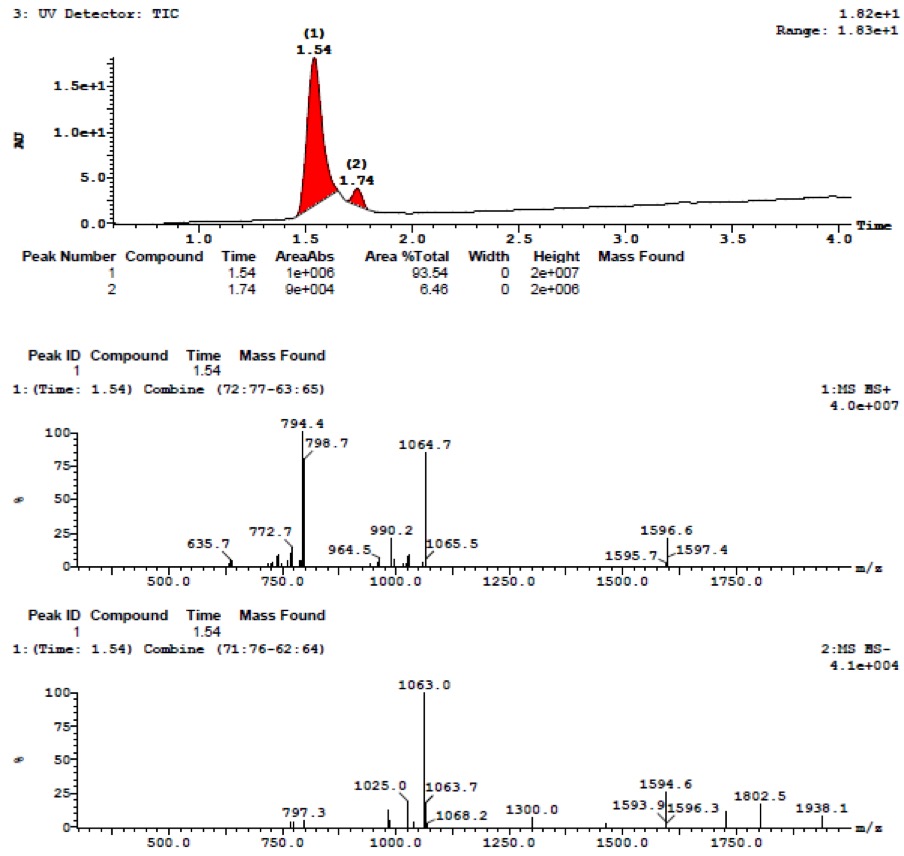


**Ppen**


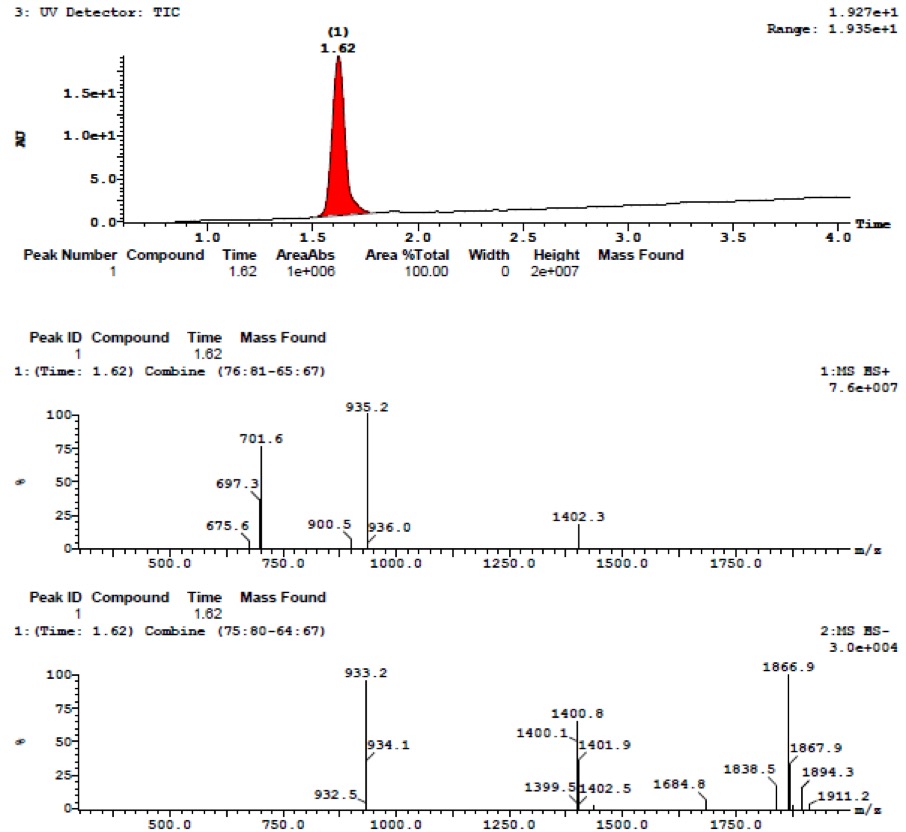

Supplement: Supplementary file 4 — Supplementary Data 1 [file 42003_2021_1736_MOESM4_ESM.docx]
