# Supplementary material for: Human ACE2 peptide-mimics block SARS-CoV-2 pulmonary cells infection
Source: Commun Biol. 2021 Feb 12;4:197. doi: 10.1038/s42003-021-01736-8 (PMC7881012; doi:10.1038/s42003-021-01736-8)
Supplement: Supplementary file 5 — Supplementary Data 2 [file 42003_2021_1736_MOESM5_ESM.docx]

**Supplementary Data 2: Materials and buffers used for BLI experiments with their corresponding suppliers and reference codes**

- SARS-CoV-2 Spike RBD Protein Fc tagged expressed in HEK293 cells (Sanyou Bio #PNA003)
- Human ACE2 Protein His tagged expressed in HEK293 cells (Sanyou Biopharmaceutical #PHA002)
- Anti-human IgG Fc capture (AHC) Biosensors (ForteBio #18-5060)
- Microplate, 96 wells, PS, black (Greiner #655209)
- DPBS+/+ (Gibco #14040091)
- Tween20 (Sigma #P7949)
- BSA: Jackson (Interchim #001-000-173)
- Running buffer: DPBS (Potassium Chloride 2.6mM, Potassium Phosphate monobasic 1.5mM, Sodium Chloride 138mM, Sodium Phosphate dibasic 8mM), 0.05% Tween 20 and 0.5% bovine serum albumin
