# Supplementary material for: Human ACE2 peptide-mimics block SARS-CoV-2 pulmonary cells infection
Source: Commun Biol. 2021 Feb 12;4:197. doi: 10.1038/s42003-021-01736-8 (PMC7881012; doi:10.1038/s42003-021-01736-8)
Supplement: Supplementary file 7 — Reporting Summary [file 42003_2021_1736_MOESM7_ESM.pdf]

nature research

Corresponding author(s): Philippe KAROYAN

Last updated by author(s): 04/01/2021

Reporting Summary

Nature Research wishes to improve the reproducibility of the work that we publish. This form provides structure for consistency and transparency in reporting. For further information on Nature Research policies, see our Editorial Policies and the Editorial Policy Checklist.

Statistics

For all statistical analyses, confirm that the following items are present in the figure legend, table legend, main text, or Methods section.

n/a	Confirmed	
☐	☒	The exact sample size (*n*) for each experimental group/condition, given as a discrete number and unit of measurement
☐	☒	A statement on whether measurements were taken from distinct samples or whether the same sample was measured repeatedly
☒	☐	The statistical test(s) used AND whether they are one- or two-sided<br>*Only common tests should be described solely by name; describe more complex techniques in the Methods section.*
☐	☒	A description of all covariates tested
☒	☐	A description of any assumptions or corrections, such as tests of normality and adjustment for multiple comparisons
☐	☒	A full description of the statistical parameters including central tendency (e.g. means) or other basic estimates (e.g. regression coefficient) AND variation (e.g. standard deviation) or associated estimates of uncertainty (e.g. confidence intervals)
☒	☐	For null hypothesis testing, the test statistic (e.g. *F*, *t*, *r*) with confidence intervals, effect sizes, degrees of freedom and *P* value noted<br>*Give P values as exact values whenever suitable.*
☒	☐	For Bayesian analysis, information on the choice of priors and Markov chain Monte Carlo settings
☒	☐	For hierarchical and complex designs, identification of the appropriate level for tests and full reporting of outcomes
☒	☐	Estimates of effect sizes (e.g. Cohen's *d*, Pearson's *r*), indicating how they were calculated

Our web collection on statistics for biologists contains articles on many of the points above.

Software and code

Policy information about availability of computer code

Data collection	NA
Data analysis	The predictive data supporting our work was generated using publicly available software. For sequence alignment, the Weblogo software (available at https://weblogo.berkeley.edu) and Clustal Omega (available at https://ebi.ac.uk/Tools/msa/clustalo/) were used. The Agadir helical content predictor is available at https://agadir.crg.es. Peptide antigenicity can be determined at https://imed.med.ucm.es/Tools/antigenic.pl. Online analysis for peptide CD spectra and using DichroWeb server and the CDSSTR algorithm is available at https://dichroweb.cryst.bbk.ac.uk. The HELIQUEST web server used to calculate peptide hydrophobicity and hydrophobic moment is available at https://heliquest.ipmc.cnrs.fr.

For manuscripts utilizing custom algorithms or software that are central to the research but not yet described in published literature, software must be made available to editors and reviewers. We strongly encourage code deposition in a community repository (e.g. GitHub). See the Nature Research guidelines for submitting code & software for further information.

Data

Policy information about availability of data

All manuscripts must include a data availability statement. This statement should provide the following information, where applicable:

- Accession codes, unique identifiers, or web links for publicly available datasets
- A list of figures that have associated raw data
- A description of any restrictions on data availability

The complex between hACE2 and the surface spike protein of SARS-CoV-2 data used for peptide design is available in RCSB PDB with the identifier doi: 10.2210/pdb6M0J/pdb.14 The randomly selected RBD amino-acid sequences from China (QOH25833, QIG55857, QHR63290, QHR63250, QJG65957, QJG65956, QJG65951), USA (QIV65044, QJD23847, QJU11481, QJD24531, QJD25193, QJD25529, QJA17180), and France (QJT73034, QJT73010, QJT72902, QJT72806, QJT72794, QJT72722,

QJT72710, QJT72626, QJT72614, QJT72554), used for sequence alignment are available in the Genebank database at ncbi.nlm.nih.gov. The amino acid sequence of the Fc-tagged 2019-nCoV RBD-SD1 was provided by Sanyou bio with purchase (sanyoubio.com/EN/2019-nCoV.php) and is depicted in Supplementary Figure 1a.

Field-specific reporting

Please select the one below that is the best fit for your research. If you are not sure, read the appropriate sections before making your selection.

- [x] Life sciences
- [ ] Behavioural & social sciences
- [ ] Ecological, evolutionary & environmental sciences

For a reference copy of the document with all sections, see nature.com/documents/nr-reporting-summary-flat.pdf

Life sciences study design

All studies must disclose on these points even when the disclosure is negative.

Sample size	All cytotoxicity experiments were measured by triplicate in three independent experiments.
Data exclusions	No values were excluded from any analysis.
Replication	Cell cytotoxicity was evaluated qualitatively by microscopic observations (not shown) and quantitatively by two different means: MTT to evaluate cell viability loss, and Annexin-V/PI staining to evaluate cell death at the highest doses used in MTT assays.
Randomization	N/A for cytotoxicity experiments.
Blinding	LGM was not aware of the chemical or biological characteristics of the peptides used when cell cytotoxicity was tested.

Reporting for specific materials, systems and methods

We require information from authors about some types of materials, experimental systems and methods used in many studies. Here, indicate whether each material, system or method listed is relevant to your study. If you are not sure if a list item applies to your research, read the appropriate section before selecting a response.

Materials & experimental systems

n/a	Involved in the study	
☐	☐	Antibodies
☐	☒	Eukaryotic cell lines
☐	☐	Palaeontology and archaeology
☐	☐	Animals and other organisms
☐	☐	Human research participants
☐	☐	Clinical data
☐	☐	Dual use research of concern

Methods

n/a	Involved in the study	
☐	☐	ChIP-seq
☐	☒	Flow cytometry
☐	☐	MRI-based neuroimaging

Antibodies

Antibodies used	NA
Validation	NA

Eukaryotic cell lines

Policy information about cell lines

Cell line source(s)	Calu-3 (ATCC HTB55) and Vero-E6 (ATCC CRL-1586) cells were purchased from the American Type Culture Collection
Authentication	BY ATCC
Mycoplasma contamination	CALU-3 cell line was tested negative for mycoplasma contamination.
Commonly misidentified lines (See ICLAC register)	NA

Palaeontology and Archaeology

Specimen provenance	NA

Specimen deposition	NA
Dating methods	NA

☐ Tick this box to confirm that the raw and calibrated dates are available in the paper or in Supplementary Information.

Ethics oversight	NA

Note that full information on the approval of the study protocol must also be provided in the manuscript.

Animals and other organisms

Policy information about studies involving animals; ARRIVE guidelines recommended for reporting animal research

Laboratory animals	NA
Wild animals	NA
Field-collected samples	NA
Ethics oversight	NA

Note that full information on the approval of the study protocol must also be provided in the manuscript.

Human research participants

Policy information about studies involving human research participants

Population characteristics	The patient recruited for virus isolation and culture was in intensive care unit
Recruitment	The patient recruited in the Pitié Salpêtrière hospital underwent a bronchoalveolar lavage for clinical purpose (seeking for a bacterial pulmonary infection)
Ethics oversight	The protocol was approved by our institution's ethics committee (Immuno-COVID-REA, CER-Sorbonne Université, no. CER-SU-2020-31).

Note that full information on the approval of the study protocol must also be provided in the manuscript.

Clinical data

Policy information about clinical studies

All manuscripts should comply with the ICMJEguidelines for publication of clinical research and a completedCONSORT checklist must be included with all submissions.

Clinical trial registration	NA
Study protocol	NA
Data collection	NA
Outcomes	NA

Dual use research of concern

Policy information about dual use research of concern

Hazards

Could the accidental, deliberate or reckless misuse of agents or technologies generated in the work, or the application of information presented in the manuscript, pose a threat to:

No	Yes	
☒	☐	Public health
☒	☐	National security
☒	☐	Crops and/or livestock
☒	☐	Ecosystems
☒	☐	Any other significant area

Experiments of concern

Does the work involve any of these experiments of concern:

No	Yes	
☒	☐	Demonstrate how to render a vaccine ineffective
☒	☐	Confer resistance to therapeutically useful antibiotics or antiviral agents
☒	☐	Enhance the virulence of a pathogen or render a nonpathogen virulent
☒	☐	Increase transmissibility of a pathogen
☒	☐	Alter the host range of a pathogen
☒	☐	Enable evasion of diagnostic/detection modalities
☒	☐	Enable the weaponization of a biological agent or toxin
☒	☐	Any other potentially harmful combination of experiments and agents

ChIP-seq

Data deposition

☐ Confirm that both raw and final processed data have been deposited in a public database such as GEO.

☐ Confirm that you have deposited or provided access to graph files (e.g. BED files) for the called peaks.

Data access links<br>*May remain private before publication.*	NA
Files in database submission	NA
Genome browser session<br>(e.g. UCSC)	NA

Methodology

Replicates	NA
Sequencing depth	NA
Antibodies	NA
Peak calling parameters	NA
Data quality	NA
Software	NA

Flow Cytometry

Plots

Confirm that:

☐ The axis labels state the marker and fluorochrome used (e.g. CD4-FITC).

☐ The axis scales are clearly visible. Include numbers along axes only for bottom left plot of group (a 'group' is an analysis of identical markers).

☐ All plots are contour plots with outliers or pseudocolor plots.

☐ A numerical value for number of cells or percentage (with statistics) is provided.

Methodology

Sample preparation	For cell death analyses by FACS, cell cultures were harvested in individual microcentrifuge tubes, centrifuged at 0.4xG 10 min, and supernatants were discarded. Cell pellets were stained with Annexin-V-APC (AnnV; 0.1 µg/mL; BD Biosciences) and propidium iodide (PI; 0.5 µg/mL; Sigma-Aldrich) in annexin binding buffer (10 mM Hepes pH 7.4, 140 mM NaCl, 2.5 mM CaCl2) and incubated in ice for 15 min.
Instrument	AnnV/PI staining was measured in a BD FACScalibur Flow cytometer.
Software	Cell death was analyzed using FlowJo software (V10.0.8).

Cell population abundance | Cell death was evaluated in 10^4 cells per sample.

Gating strategy | Cell populations were distinguished from debris in untreated controls. Annexin-V-APC (FLH-4) and PI (FLH-2) positivity of cell populations was set at the beginning of the exponential increase of fluorescence.

☐ Tick this box to confirm that a figure exemplifying the gating strategy is provided in the Supplementary Information.

Magnetic resonance imaging

Experimental design

Design type: NA

Design specifications: NA

Behavioral performance measures: NA

Acquisition

Imaging type(s): NA

Field strength: NA

Sequence & imaging parameters: NA

Area of acquisition: NA

Diffusion MRI ☐ Used ☒ Not used

Preprocessing

Preprocessing software: NA

Normalization: NA

Normalization template: NA

Noise and artifact removal: NA

Volume censoring: NA

Statistical modeling & inference

Model type and settings: NA

Effect(s) tested: NA

Specify type of analysis: ☐ Whole brain ☐ ROI-based ☐ Both

Statistic type for inference (See Eklund et al. 2016): NA

Correction: NA

Models & analysis

n/a	Involved in the study
☒	☐ Functional and/or effective connectivity
☒	☐ Graph analysis
☒	☐ Multivariate modeling or predictive analysis
